# Physical and Physiological Monitoring on Red Palm Weevil-Infested Oil Palms

**DOI:** 10.3390/insects11070407

**Published:** 2020-06-30

**Authors:** Nazmi Harith-Fadzilah, Mohamad Haris-Hussain, Idris Abd Ghani, Azlina Zakaria, Samsudin Amit, Zamri Zainal, Wahizatul Afzan Azmi, Johari Jalinas, Maizom Hassan

**Affiliations:** 1 Institute of Systems Biology, Universiti Kebangsaan Malaysia, Bangi 43600, Selangor, Malaysia; P92950@siswa.ukm.edu.my (N.H.-F.); zz@ukm.edu.my (Z.Z.); 2 Department of Biological Sciences and Biotechnology, Faculty of Science and Technology, Universiti Kebangsaan Malaysia, Bangi 43600, Selangor, Malaysia; p100206@siswa.ukm.edu.my (M.H.-H.); idrisyatie@ukm.edu.my (I.A.G.); johari_j@ukm.edu.my (J.J.); 3 Sime Darby Research Sdn. Bhd., KM10, Jalan Banting-Kelanang, Banting 42700, Selangor, Malaysia; azlina.zakaria@simedarbyplantation.com (A.Z.); samsudin.amit@simedarbyplantation.com (S.A.); 4 Faculty of Science and Marine Environment, Universiti Malaysia Terengganu, Kuala Nerus 21030, Terengganu, Malaysia; wahizatul@umt.edu.my

**Keywords:** *Rhynchophorus ferrugineus*, red palm weevil, oil palm, *Elaeis guineensis*, herbivory, photosynthesis, insect-plant interactions

## Abstract

The red palm weevil (RPW) is a stem boring Coleoptera that decimates host palm trees from within. The challenge of managing this pest is due to a lack of physical symptoms during the early stages of infestation. Investigating the physiological changes that occur within RPW-infested palm trees may be useful in establishing a new approach in RPW detection. In this study, the effects of RPW infestation were investigated in *Elaeis guineensis* by observing changes in physical and physiological parameters during the progress of infestation by visual inspection and the comparison of growth, gas exchange, stomatal conductance, and chlorophyll content between the non-infested control, physically wounded, and RPW-infested *E. guineensis* groups. During the study period, four distinct levels of physical infestation were observed and recorded. The RPW-infested group displayed significantly lower maximum photosynthesis activity (A_max_) starting from the third week post-infestation. However, growth in terms of change in plant height and stem circumference, leaves’ stomatal conductance, and chlorophyll content were not significantly different between the three groups during the duration of the study. The significant drop in photosynthesis was observed one week before physical changes appeared. This suggests the promising utilisation of photosynthesis activity as a signal for detecting RPW infestation at the early stage of attacks, which could be useful for integration in integrated pest management (IPM).

## 1. Introduction

The red palm weevil (RPW), *Rhynchophorus ferrugineus* (Olivier, 1790), is a destructive and invasive stem-boring pest affecting the majority of palm species worldwide. The RPW in Malaysia is believed to have originated from the Middle Eastern countries [1]. In the South East Asian region, cases of RPW infestation have been reported in Thailand, Malaysia, Indonesia, the Philippines, India, and Sri Lanka where economically important palm species such as coconut (*Cocos nucifera*) and oil palm (*Elaeis guineensis*), as well as ornamental palms such as ribbon fan palm and Chinese fan palm, are affected [2]. RPW is at its most damaging during the larval stage. The eggs hatch inside the cracks and crevices of the soft tissue of the palm, primarily at the crown of the host palm [3,4,5]. From here, the larvae chew the soft tissues, gradually creating deep galleries into the tree’s structure. It is difficult to identify infested palm trees in palm plantations due to the lack of obvious symptoms of infestation, especially at the early stage of infestation. As the infestation becomes more severe, physical signs become more apparent [6].

Alternative methods of infestation detection are being explored with the objective of detecting infested palms at an early stage. Some examples include using trained sniffing dogs and monitoring acoustic signals attributed to RPW larva chewing [7,8]. However, each alternative had issues in terms of poor accuracy or limited feasibility for large-scale plantations. Thus, the conventional mean of detection remains relying on discerning physical symptoms of RPW infestation on palm trees. Physical symptoms of infestation have been well-documented for the case of *C. nucifera*, *Phoenix dactylifera*, and *P. canariensis*. Güerri-Agulló et al. [9] categorised the stages of RPW infestation into five levels based on the severity of the infestation observed in *P. canariensis*. Currently, the most reliable monitoring method for detecting RPW infestation is via visual inspection by growers [6,10]. However, the process is often time-consuming and visible only when the infestation of RPW has become severe.

Recent studies have explored the impact of RPW infestation on the host’s physiology. It is established that the insect herbivory activity can induce physiological changes in terms of growth, temperature, photosynthetic activity, fruit yield, and reproductive potential [11,12]. Change in physiology such as temperature and photosynthesis activity can be easily measured with the current devices such infra-red cameras and gas exchange, which are portable and provide rapid measurements [13,14]. However, the physiological impacts on the host plant are dependent on the invading insects and host types. Stem-borer insects’ herbivory does not appear to have consistent effects on the host plants. A previous investigation on the impact of RPW infestation on *P. canariensis* found that the infested palm had significantly lower stomatal conductance [10]. However, another study on the impact of RPW infestation on *P. dactlyifera* reported that the stomatal conductance was not impacted by RPW larvae herbivory [15]. Thus, it is worth investigating the impact of RPW on other host palms as the different host species may have different physiological responses.

To our knowledge, there are no studies that monitor the progression of the infestation of RPW on the palm host, both physically and physiologically. In this study, we performed artificial infestation of RPW on *E. guineensis* and subsequently monitored the progression of the physical signs of infestation and also the physiological changes during the infestation period in terms of growth and photosynthesis activity. Given the nature of the RPW infestation, which decimates host palms from within, the change in infested palms’ physiology, such as photosynthesis activity, may be useful as an indication of RPW infestation. Through monitoring progression of the infestation, the timing of when both physical and physiological changes occur is compared to establish whether the physiological aspects of the RPW-infested palm can be used as an alternative to physical symptoms to give an earlier indication of RPW infestation.

## 2. Materials and Methods 

### 2.1. RPW Rearing

Adult RPWs were sampled by the Terengganu Department of Agriculture from heavily-infested coconut plantations in Terengganu by using specific pheromone lures (4-methyl-5-nonanol) and pheromone traps [1,16,17]. Adult RPWs were reared in covered plastic containers (10 cm diameter × 7 cm high) and kept under laboratory conditions (25 ± 2 °C, 70 ± 5% RH, photoperiod of 12:12 (L:D) h) with chopped sago palm stems as a source of food [18]. Adults were allowed to mate and the eggs were collected and placed in a sterile 9-cm-diameter plastic Petri dish containing a self-made artificial diet substrate based on standard methods and ingredients (5% *w*/*v* of yeast extract, 5% *w*/*v* of wheat germ, 5% *w*/*v* of corn meal, 1.2% *w*/*v* of vitamin C, 1.5% *w*/*v* of bacteriological agar, 0.15% *w*/*v* of ascorbic acid, 0.18% *w*/*v* of ethyl-4-hydroxybenzoate, 0.18% *w*/*v* of benzoic acid, and 0.05% *w*/*v* of chloramphenicol) [19,20]. RPW larvae were reared on the artificial diet until they reached 30-d of age [20]. To maintain the humidity of rearing containers, filter paper (20 by 5 cm) dampened with distilled water was placed below the specimen containers [20].

### 2.2. E. Guineensis Infestation with RPW Larva

Two-year-old tissue cultured *E. guineensis* plantlings of Dura cultivars were obtained from Sime Darby *E. guineensis* plantation. The experiment was conducted in an experimental plot at Universiti Kebangsaan Malaysia (UKM), NE (2°55′ lat 101°47′ long). The plot is an enclosure covered by steel mesh screens as to contain the RPW-infested *E. guineensis* while exposing all plantlings to the ambient climate. The relative humidity and ambient temperature were in the range of 50.5–64% and 34.1–38.8 °C, respectively. Eighteen *E. guineensis* palm plantlings were divided into three groups (control, wounded, and infested) with six replicates for each group. The choice of the sample size was due to the physical space constraints of the enclosure. The RPW is a very destructive pest, so strict containment was necessary. Furthermore, this practice was based on a previous study investigating the impact of RPW infestation on *P. dactylifera* [21]. For the control group, *E. guineensis* were allowed to grow normally. For both wounded and infested groups, three distinct holes (1.5-cm-diameter by 5-cm depth) were drilled at the crown of each plantlings by using a power drill with a 1.5-cm-diameter size bit [20]. For the infested group, three 30-d old RPW larvae were introduced artificially into each hole. The number of larva introduced was less than previous research to account for the small palm size and thickness used in this study [21]. Subsequently, the holes from *E. guineensis* wounded and infested group were closed by *E. guineensis* tissues before being wrapped with clear plastic wrapper. All the *E. guineensis* plantlings were placed inside the closed and sealed greenhouse to prevent other insects from entering the study site. Any attack symptoms and changes on the *E. guineensis* specimens were observed and recorded weekly. The guidelines for classifying the level of RPW infestations were based on previous studies conducted on other palm species such as *P. canariensis* and *C. nucifera* [5,9]. Four levels of infestation were recorded, which were: Level 1 (no visual symptoms), Level 2 (larval feeding signs), Level 3 (loss of leaf symmetry at the palm upper crown), and Level 4 (palm shows no leaf at the upper crown).

### 2.3. Plantling Growth Measurement

*E. guineensis* growth in each group was assessed for change in height and circumference. Plant height was measured from the base of the stem to the end of the rachis of the tallest frond [22]. Plant circumference was measured at 5 cm from the base of the stem [23]. Plant height and circumference were measured on the first day post-infestation by RPW and the ninth week when the severe collapse of palm frond in the infested group was observed. Growth was calculated as follows:

Growth (cm) = height/circumference of *E. guineensis* on Week 9 − height/circumference of *E. guineensis* on Day 1.

### 2.4. Photosynthetic Activity Analysis

Measurement of maximum photosynthesis rate (A_max_) and stomatal conductance were measured and calculated on *E. guineensis* intact leaves using an infrared gas analyser system (LI-6400, LI-COR Inc., Lincoln, NE, USA) at optimal cuvette settings (photon flux density of 1000 µmol photosynthetically active radiation m^2^s^−1^; 400 ppm CO_2_, 30 °C leaf temperature) [24]. Measurements were taken at the ninth frond as the ninth frond was at the middle of the crown where the youngest mature leaves can be found and its position allows for a more representative measurement of the overall photosynthesis activity of *E. guineensis* [24]. Measurements were taken at first day after infestation, followed by weeks 1, 3, 4, 6, 8, and 9, when the infestation had reached Level 4.

### 2.5. Chlorophyll Content Analysis

Leaf sampling was performed on the eighth and tenth frond of *E. guineensis* from the control, wounded, and infested group at 1, 3, and 6 weeks after RPW infestation. Three plantlings from each group were randomly selected and for each plantling, five leaf blades were snipped randomly from the eighth and the tenth frond. Each snipped leaf blade was ensured that only the first 15 cm from the base of the leaf was collected. Chlorophyll content from *E. guineensis* leaves were determined by an established method from Ni et al. [25]. Absorbance measurement was taken at 663 nm (A663) and 645 nm (A645). The chlorophyll concentration was calculated as:

$$\mathrm{Chlorophyll}\;\mathrm{concentration}\;\left(\mathrm{mg}/g\right)=\left[8.02\;{*\;A}_{663}+20.20\;{*\;A}_{645}\right]\;*\;\frac{V}{1000\;}*\;W\;$$

where V = volume of extract (mL); W = weight of fresh leaves (g).

### 2.6. Statistical Analysis

All statistical analyses were performed using SPSS statistical software version 25 (SPSS, Chicago, IL, USA). The Shapiro–Wilk test was carried out for all analysis to assess normality. One-way analysis of variance (ANOVA) followed by Tukey’s Honest Significant Difference (HSD) test was used to identify the statistically significant difference of readings among the control, wounded, and infested groups for aspects of growth, photosynthetic activity, stomatal conductance, and chlorophyll content. Two-way ANOVA following post-hoc tests were used to assess the interaction between the experimental groups and time-post infestation.

## 3. Results

### 3.1. Symptoms of Infestation

**Figure 1:**
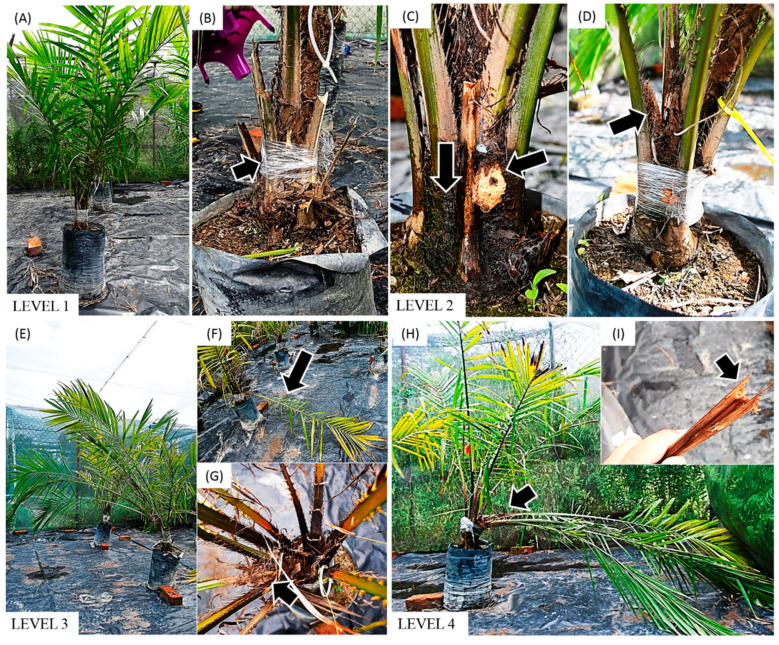
Visual symptom level of RPW infestation in oil palm plantlings based on empirical observations in this greenhouse experiment. (**A**,**B**) Level 1 infestation observed between first to the third week: no symptoms visible especially at the sealed crown base. (**C**,**D**) Level 2 infestation observed between fourth to sixth week: dark oozes and smelly sap together with soft, creamy-coloured tissue appeared at the crown base; brown sawdust recorded between the plantling’s trunk and the base of the leaves. (**E**–**G**) Level 3 infestation observed between sixth to ninth week: host plant’s shoot started to lean to the ground; the leaves started to fall; loss of symmetry at palm upper crown. (**H**,**I**) Level 4 infestation observed at the ninth week: leaves collapsed and started to show skirting shape; shoot was dried out and fell off.

The infestation by RPW occurred at a rapid rate. Within the period of nine weeks, the infestation had progressed from initiation (Level 1) to irreparable damage (Level 4) (Table 1). The shift between each infestation stage took approximately three weeks, i.e., Level 1 to Level 2 (first to third week), Level 2 to Level 3 (fourth to sixth week), and Level 3 to Level 4 (seventh to ninth week) (Figure 1).

During Level 1 infestation, there were no visual symptoms of attack by the RPW larvae observed on the infested *E. guineensis* group (Figure 1A,B). At the fourth week, symptoms of dark and smelly sap oozes, the protrusion of soft, creamy-coloured tissue fibres, and a sawdust appearance near the palm base, which is associated with Level 2 infestation, began to appear (Figure 1C,D). Level 3 infestation started to appear in the seventh week, characterised by the loss of leaf symmetry at the palm’s upper crown due to frond collapse (Figure 1E–G). This visible symptom progressed to Level 4 in the ninth week, where the shoot turned brown and dried, while all the leaves collapsed due to the broken base of the leaves. Here, the infested *E. guineensis* appeared umbrella-shaped (Figure 1H,I).

The fast infestation progression exhibited by RPW was previously reported. Salem (2015) reported that RPW larvae were capable of causing irreparable damage to date palm trees (Level 4) within two months after infestation, which is in accordance with our findings [7]. In addition, the symptoms that appeared in *E. guineensis* in this study displayed similar characteristics with those from RPW-infested *P. canariensis* and *P. dactylifera* [9]. This suggests that the physical characteristics of RPW infestation made from this study could be extrapolated as guide for monitoring the stage of infestation for the other palm species.

### 3.2. Effect of RPW Infestation on Plant Growth Performance

**Figure 2:**
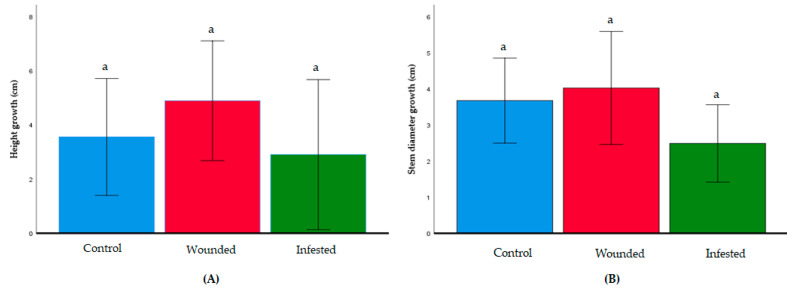
Growth of each *E. guineensis* group. (**A**) Growth in terms of increase in palm height; (**B**) growth in terms of increase in stem circumference. Data shown as mean + S.D. No significant differences among the control, wounded, and infested group were observed based on analysis of variance (ANOVA) following the Tukey multiple comparison test (*p* > 0.05).

The average growth of *E. guineensis* in the control, wounded, and infested groups was compared in terms of change in plant height and stem thickness. Over the span of nine weeks, the infested group showed no significant growth differences compared to the wounded and control groups (*p* < 0.05) for both parameters (Figure 2). The control, wounded, and infested groups had an average plant height growth of 3.78 cm, 4.02 cm, and 2.48 cm, respectively. For stem circumference, the three groups had growth of 8.58 cm, 7.55 cm, and 4.9 cm, respectively.

### 3.3. Effect of RPW Infestation on Plant Photosynthesis

**Figure 3:**
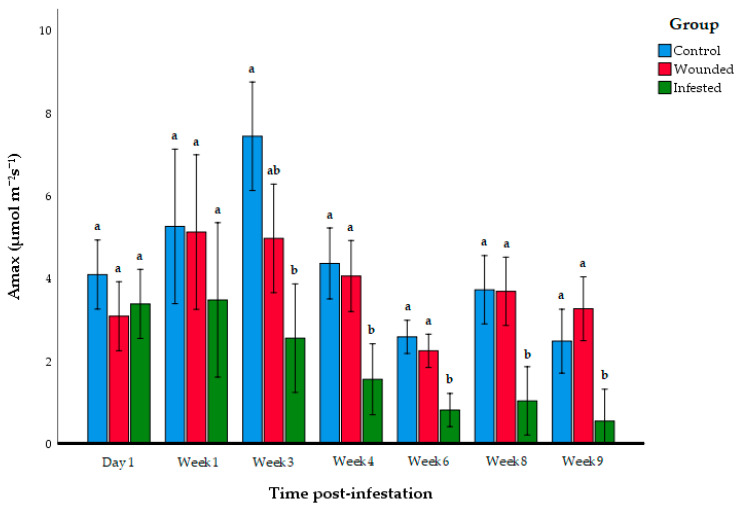
Mean maximum photosynthesis rate (A_max_) of each *E. guineensis* group at different time post-infestation. Data shown as mean + S.D. Different letters indicate significant differences among the control, wounded, and infested group at each time post-infestation period based on ANOVA following the Tukey multiple comparison test (*p* > 0.05).

**Figure 4:**
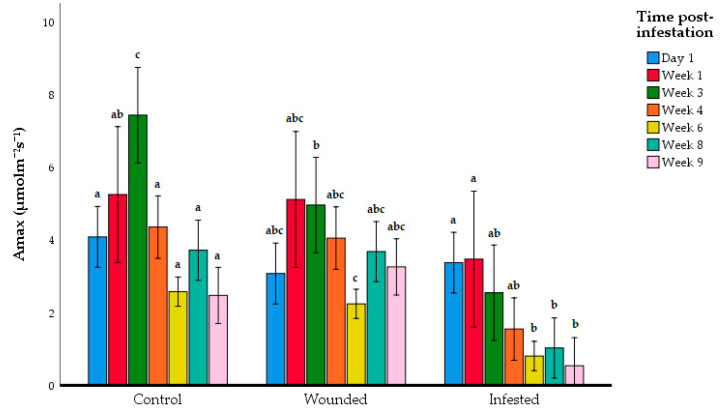
Mean maximum photosynthesis rate (A_max_) of *E. guineensis* sorted by experimental group. Data shown as mean + S.D. Different letters indicate significant differences among post-infestation times within each experimental group based on ANOVA following the Tukey multiple comparison test (*p* > 0.05).

RPW larvae herbivory began to show an impact on photosynthesis at the beginning of the third week post-infestation. On the third week after infestation, the A_max_ of the infested *E. guineensis* group began to show a significant reduction compared to the control group, but it was not significantly lower than the wounded group (Figure 3, Table 2). However, on the fourth week, the infested *E. guineensis* group began to display significantly lower A_max_ than both the control and wounded group. This reduction in photosynthesis activity of the RPW-infested *E. guineensis* continued until the ninth week, where the RPW infestation had entered Level 4 (Figure 1). ANOVA showed a significant interaction between the experimental groups and time post-infestation (*F*_6,105_ = 14.8, *p* = 0.005), so Tukey’s post-hoc test was also employed to compare the A_max_ values between the time post-infestation for each experimental group (Figure 4). Indeed, the A_max_ reading was significantly higher on Week 3 compared to the other infestation periods for the control. In contrast, there was a significant difference between the Week 3 and Week 6 A_max_ values in wounded group. Overall, there was no clear correlation between the photosynthesis activity and time-post infestation for the control and wounded group. In contrast, there was an observable correlation between the decline of photosynthetic activity and the progression of the time post-infestation in the infested group. The Day 1 and Week 1 A_max_ measurements showed the instances of the Level 1 infestation. In the period where the physical infestation advanced to Level 2 (Week 3 to Week 6), the A_max_ values were at the intermediate levels between Day 1 until Week 1, and Week 6 until Week 9. At Level 3 and Level 4 infestation, the A_max_ levels were already significantly lower than during Level 1 infestation.

**Figure 5:**
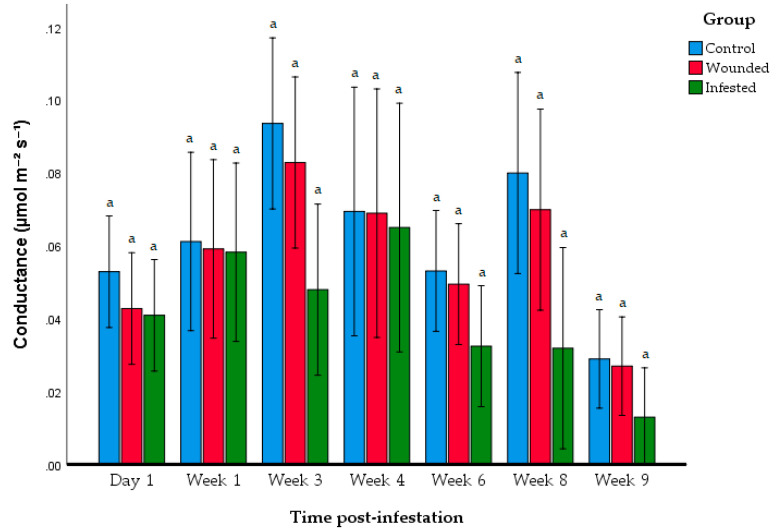
Stomatal conductance of each group at at different periods after infestation. Data shown as mean + S.D. Different letters indicate significant differences among the control, wounded, and infested group within each time post-infestation period based on ANOVA following the Tukey multiple comparison test (*p* > 0.05).

**Figure 6:**
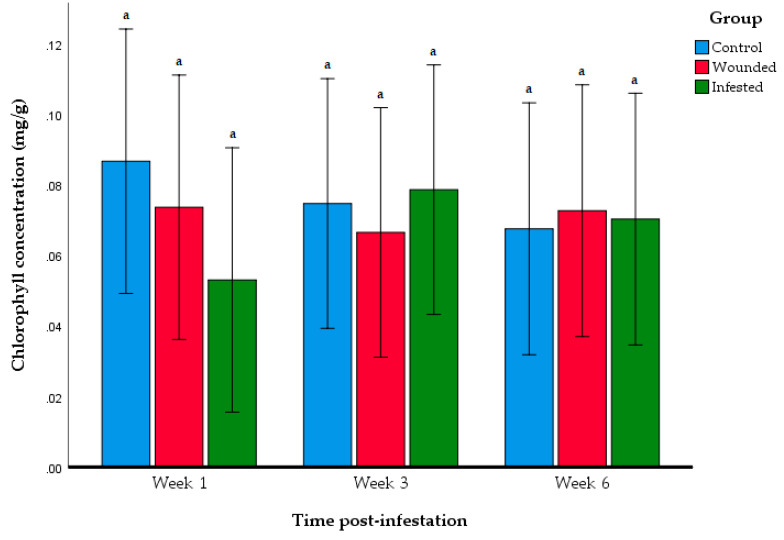
Total chlorophyll content of *E. guineensis* in each group at different periods after infestation. Data shown as mean + S.D. Different letters indicate significant differences among the control, wounded, and infested group based on ANOVA following the Tukey multiple comparison test (*p* > 0.05).

Further investigation of photosynthesis activity was performed through stomatal conductance and total chlorophyll content. The stomatal conductance levels were not significantly different between the control, wounded, and infested groups over the span of nine weeks (Figure 5, Table 3). No significant interaction was found between the experimental groups and time post-infestation (*F*_12,105_ = 0.841, *p* = 0.608) in terms of stomatal conductance. Total chlorophyll content was compared between the three groups on Week 1, 3, and 6 (Figure 6). No significant differences were observed between the control, wounded, and infested group for all three weeks. 

## 4. Discussion

The present study showed the order of appearance of physical damage on the *E. guineensis* host as the RPW infestation progressed from the initial stage to the terminal stage. The wounded group is included in the investigation as to monitor the possible effect of physical wounding without insect herbivory could have on *E. guineensis*. The rate of RPW infestation progressed in a relatively rapid rate. Physical symptoms first appeared beginning the fourth week of post-infestation. Just three weeks after that, significant damage was inflicted upon the *E. guineensis* host where signs of frond collapse appeared. Although the infestation did not appear to affect growth performance, it negatively impacted the photosynthetic activity of the host. However, the stomatal conductance and chlorophyll content were not affected by the infestation. This suggests that the infestation by RPW larvae did not impact the morphological function of the photosynthesis mechanisms in leaves.

RPW larvae achieved severe damage in the host *E. guineensis* within only the span of nine weeks. *E. guineensis* is a perennial plant with slow growth rate [26]. The progression of infestation from early (Level 1) to severe (Level 4) by RPW larvae was too rapid that its impact on host *E. guineensis* growth could not be observed. However, it must be noted that this is an infestation on young oil palm plantlings. Due to its significantly smaller stem thickness, the impact of RPW infestation could be exacerbated. It can be speculated that infestation on more mature hosts would take much longer time to reach Level 4.

Furthermore, the physiological changes to the infested *E. guineensis* were observed earlier than the physical symptoms of attacks. Namely, the photosynthetic activity significantly dropped at Week 3 when the infestation was still in Level 1 and where no physical symptoms of infestation could be observed (Figure 1). This finding is within the expected outcome as biotic stresses induce metabolic shifts from productivity (e.g., photosynthesis, growth) to survival (e.g., production of antioxidants, plant hormones) in plants [27,28]. Thus, it might be possible that the decline in photosynthetic activity observed in the infested *E. guineensis* was the consequence of the genes and proteins relating to photosynthesis being downregulated. Consequently, these physiological changes can occur earlier than the appearance of physical symptoms of stem borer herbivory.

In addition, a gradual decline in A_max_ of the infested *E. guineensis* group could also be observed as the infestation progressed (Figure 4). This suggests a correlation between the photosynthesis activity of the host palm and severity of RPW larvae infestation. However, it was noted that the physical symptoms did not progress in a similar fashion. Some infested *E. guineensis* showed physical symptoms earlier than the other plantlings. 

Furthermore, the correlation between the photosynthesis activity of host plant and the severity of the RPW-infestation was in contrast with a previous study observing the photosynthesis activity of RPW-infested *P. dactylifera* palms [15]. In that study, the RPW-infested *P. dactylifera* palms at medium severity had significantly lower photosynthesis, but for severely infested palms, the level of photosynthesis activity was comparable to non-infested palms. This difference in observation may likely be due to differences in ambient climate, the condition of the soil, and the experimental design as well. In this study, *E. guineensis* were watered every two days to minimise any difference in photosynthetic activity that might be caused by water deficiency in soil. Previous investigations have reported that water deficiency can induce stress that impairs photosynthesis activity. Water-deficient stress leads to significantly lower photosynthetic activity, stomatal conductance, and chlorophyll content in *E. guineensis* [29,30]. Thus, by watering the plantlings, it can be certain that the lower rate of photosynthesis observed in the infested *E. guineensis* was due to RPW herbivory rather than the environmental conditions at the time. 

The contrasting observations between the A_max_ levels and both stomatal conductance and chlorophyll content might be attributable to the nature of the RPW infestation itself. Al-Jabr et al. (2007) showed that there was a poor correlation between intercellular carbon dioxide concentration and stomatal conductance due to RPW herbivory [15]. The author inferred that RPW herbivory does not damage leaf morphology. Consequently, the photosynthesis-related structures of the host’s leaves would remain intact and functional. This observation might rather be attributed to the systemic acquired resistance (SAR) of *E. guineensis* being active rather than the photosynthesis machinery being impaired. Under the event of insect herbivory or pathogen attacks, host plants begin producing phytohormones and other signalling molecules that travel throughout the plant and stimulated the production of defence-related proteins and metabolites [31,32]. Among the resulting effects of SAR is the downregulation of expression of genes and proteins related to photosynthesis. Thus, the effect of decline in photosynthetic activity of the infested *E. guineensis* might be attributable to the SAR response by *E. guineensis* to resist infestation by RPW. It might be worth exploring the genes or proteins that correlate with this observation in future research. However, the findings reported here were derived from small sample size (*n* = 6 per group), which could be subjected to large variability as observed in stomatal conductance analysis (Figure 5). This investigation was conducted as a preliminary study before conducting a larger scale study involving mature oil palms and under field conditions, and it has shown a correlation between the severity of infestation with the decline in photosynthesis activity.

## 5. Conclusions

RPW larvae herbivory inflicted severe, irreversible damage to *E. guineensis* palms within nine weeks of infestation. The progression of infestation took only nine weeks to achieve severe damage to the two-year-old host, which was far more rapid in comparison to other stem-boring Coleopterans. Apart from physical damage, the herbivory also hampered photosynthesis activity significantly. Physiological changes to the infested *E. guineensis* were observed more than the visible signs of physical damage that appeared beginning four weeks post infestation, whereas photosynthetic activity significantly dropped in the third week after infestation. However, although the photosynthesis activity was reduced, *E. guineensis* growth, stomatal conductance, and chlorophyll content were not significantly affected by the infestation. This gives a promising novel approach of identifying infested *E. guineensis* palms at an early stage of RPW infestation, which could potentially be applied as part of the integrated pest management (IPM) for managing RPW populations. However, further research is necessary for establishing a threshold photosynthesis level in mature infested *E. guineensis* palms under field settings at different ages, while additionally accounting for the effect of water deficiency as this condition can also exhibit a drop in photosynthesis. 

## Figures and Tables

**Table 1:** Classification of red palm weevil (RPW) infestation level in *E. guineensis* plantlings.

Level	Figure 1	Descriptions
1 (Early stage)	A, B	No visual symptoms
2 (Intermediate I)	C, D	Dark sap visible Tissue fibre protrusion and sawdust presence
3 (Intermediate II)	E, F, G	Initial signs of frond collapse
4 (Final stage)	H, I	Collapsed frond turned brown

**Table 2:** Mean ± SD and results of one-way ANOVA of the maximum photosynthesis rate (A_max_) at each time post-infestation. Different letters indicate significant differences based on ANOVA following Tukey multiple comparison test (*p* > 0.05).

Group	Day 1	Week 1	Week 3	Week 4	Week6	Week 8	Week 9
Control	4.19 ± 1.13 ^a^	3.66 ± 1.10 ^a^	7.44 ± 1.103 ^a^	4.362 ± 0.753 ^a^	2.583 ± 0.326 ^a^	3.724 ± 1.12 ^a^	2.48 ± 0.567 ^a^
Wounded	3.083 ± 0.90 ^a^	5.122 ± 1.09 ^a^	4.97 ± 1.91 ^a,b^	4.057 ± 1.34 ^a^	2.25 ± 0.602 ^a^	3.687 ± 0.909 ^a^	3.265 ± 1.23 ^a^
Infested	3.81 ± 1.27 ^a^	3.19 ± 1.54 ^a^	2.55 ± 1.408 ^b^	1.56 ± 0.754 ^b^	0.812 ± 0.418 ^b^	1.03 ± 0.79 ^b^	0.543 ± 0.618 ^b^
ANOVA F-value	1.74	2.35	15.7	14.6	24.8	15.8	14.9
*p*-value	0.290	0.130	2.08 × 10^−4^	3.05 × 10^−4^	1.77 × 10^−5^	2.02 × 10^−4^	2.74 × 10^−4^

**Table 3:** Mean ± SD and results of one-way ANOVA of stomatal conductance at each time post-infestation. Different letters indicate significant differences based on ANOVA following Tukey multiple comparison test (*p* > 0.05).

Group	Day 1	Week 1	Week 3	Week 4	Week6	Week 8	Week 9
Control	0.053 ± 0.005 ^a^	0.0612 ± 0.0133 ^a^	0.0936 ± 0.0151 ^a^	0.0694 ± 0.02553 ^a^	0.0531 ± 0.0153 ^a^	0.0799 ± 0.0197 ^a^	0.029 ± 0.0128 ^a^
Wounded	0.428 ± 0.233 ^a^	0.0591 ± 0.0293 ^a^	0.0828 ± 0.0359 ^a^	0.0689 ± 0.0418 ^a^	0.0195 ± 0.0220 ^a^	0.0699 ± 0.0427 ^a^	0.027 ± 0.0211 ^a^
Infested	0.049 ± 0.189 ^a^	0.0582 ± 0.0365 ^a^	0.0480 ± 0.02577 ^a^	0.065 ± 0.469 ^a^	0.0325 ± 0.0192 ^a^	0.0319 ± 0.0284 ^a^	0.0130 ± 0.00106 ^a^
ANOVA F-value	0.804	0.017	4.69	0.023	2.004	3.84	1.89
*p*-value	0.466	0.983	0.066	0.977	0.169	0.065	0.186

## References

[1] Azmi W.A., Daud S.N., Hussain M.H., Wai Y.K., Chik Z., Sajap A.S. (2014). Field trapping of adult red palm weevil *Rhynchophorus ferrugineus* Olivier (Coleoptera: Curculionidae) with food baits and synthetic pheromone lure in a coconut plantation. Philipp. Agric. Sci..

[2] Azmi W.A., Lian C.J., Zakeri H.A., Yusuf N., Omar W.B.W., Wai Y.K., Zulkefli A.N., Hussain M.H. (2017). The red palm weevil, *Rhynchophorus ferrugineus*: Current issues and challenges in Malaysia. Oil Palm Bull..

[3] Murphy S., Briscoe B. (1999). The red palm weevil as an alien invasive: Biology and the prospects for biological control as a component of IPM. Biocontrol News Inf..

[4] Rochat D., Dembilio O., Jaques J.A., Suma P., La Pergola A., Hamidi R., Kontodimas D., Soroker V. (2017). Rhynchophorus ferrugineus: Taxonomy, distribution, biology, and life cycle. Handbook of Major Palm Pests: Biology and Management.

[5] Wahizatul A.A., Zazali C., Abdul R., Nurul’Izzah A.G. (2013). A new invasive coconut pest in Malaysia: The red palm weevil (Curculionidae: *Rhynchophorus ferrugineus*). Plant. Kuala Lumpur.

[6] Kontodimas D., Soroker V., Pontikakos C., Suma P., Beaudoin-Ollivier L., Karamaouna F., Riolo P. (2016). Visual identification and characterization of *Rhynchophorus ferrugineus* and *Paysandisia archon* infestation. Handb. Major Palm Pests Biol. Manag..

[7] Salem S.A. (2015). Accuracy of trained dogs for early detection of red palm weevil, *Rhynchophorus ferrugineus Oliv.* infestations in date palm plantations. Swift J. Agric. Res..

[8] Hetzroni A., Soroker V., Cohen Y. (2016). Toward practical acoustic red palm weevil detection. Comput. Electron. Agric..

[9] Güerri-Agulló B., López-Follana R., Asensio L., Barranco P., Lopez-Llorca L.V. (2011). Use of a solid formulation of *Beauveria bassiana* for biocontrol of the red palm weevil (*Rhynchophorus ferrugineus*) (Coleoptera: Dryophthoridae) under field conditions in SE Spain. Florida Entomol. Soc..

[10] Soroker V., Suma P., Pergola A.L., Cohen Y., Akchanatis V., Golomb O., Alchanatis V., Golomb O., Goldshtein E., Hetzroni A. Early detection and monitoring of red palm weevil: Approaches and challenges. Proceedings of the AFPP—Palm Pest Mediterranean Conference.

[11] Stephens A.E.A., Westoby M. (2015). Effects of insect attack to stems on plant survival, growth, reproduction and photosynthesis. Oikos.

[12] Golomb O., Alchanatis V., Cohen Y., Levin N., Soroker V. Detection of red palm weevil infected trees using thermal imaging. Proceedings of the 10th European Conference on Precision Agriculture (ECPA 2015).

[13] Jaafar H., Ibrahim M.H. (2012). Photosynthesis and quantum yield of oil palm seedlings to elevated carbon dioxide. Advances in Photosynthesis—Fundamental Aspects.

[14] Soroker V., Suma P., Pergola A.L., Llopis V.N. (2017). Surveillance techniques and detection methods for Rhynchophorus ferrugineus and Paysandisia archon. Handbook of Major Palm Pests: Biology and Management.

[15] Al-Jabr A.M., Al-Khateeb A.A., Al-Khateeb S.A., Al-Ayied H.Y. (2007). Effects of red palm weevil *Rynchophorus ferrugineus* (Olivier) infestation on gas exchange capacity of two date palm *Phoenix dactylifera* L. cultivars. J. Biol. Sci..

[16] Hallett R.H., Oehlschlager A.C., Borden J.H. (1999). Pheromone trapping protocols for the Asian palm weevil, *Rhynchophorus ferrugineus* (Coleoptera: Curculionidae). Int. J. Pest Manag..

[17] Haris M., Nang M., Chuah T., Azmi W.A. (2014). The efficacy of synthetic food baits in capturing red palm weevil, Rhynchophorus ferrugineus (Coleoptera: Curculionidae) in campus area of Universiti Malaysia Terengganu. Serangga.

[18] Wai Y.K., Bakar A.A., Azmi W.A. (2015). Fecundity, fertility and survival of red palm weevil (*Rhynchophorus ferrugineus*) larvae reared on Sago Palm. Sains Malays..

[19] Alarcon F.J., Martınez T.F., Barranco P., Cabello T., Diaz M., Moyano F. (2015). Digestive proteases during development of larvae of red palm weevil, *Rhynchophorus ferrugineus* (Olivier, 1790) (Coleoptera: Curculionidae). Insect Biochem. Mol. Biol..

[20] Jalinas J., Güerri-Agulló B., Mankin R.W., López-Follana R., Lopez-Llorca L.V. (2015). Acoustic assessment of *Beauveria bassiana* (Hypocreales: Clavicipitaceae) effects on *Rhynchophorus ferrugineus* (Coleoptera: Dryophthoridae) larval activity and mortality. J. Econ. Entomol..

[21] Rasool K.G., Khan M.A., Tufail M., Husain M., Mehmood K., Mukhtar M., Takeda M., Aldawood A.S., Curculionidae W.C., Ghulam K. (2018). Differential proteomic analysis of date palm leaves infested with the red palm weevil (Coleoptera: Curculionidae). Florida Entomol..

[22] Rusli M.H., Idris A.S., Cooper R.M. (2015). Evaluation of Malaysian oil palm progenies for susceptibility, resistance or tolerance to *Fusarium oxysporum* f. sp.. elaeidis and defence-related gene expression in roots. Plant Pathol..

[23] Lim S.-L., Subramaniam S., Zamzuri I., Amir H.G. (2018). Growth and biochemical profiling of artificially associated micropropagated oil palm plantlets with *Herbaspirillum seropedicae*. J. Plant Interact..

[24] Haniff M.H. (2006). Gas exchange of excised oil palm (*Elaeis guineensis*) fronds. Asian J. Plant Sci..

[25] Ni Z., Kim E.-D., Chen Z.J. (2009). Chlorophyll and starch assays. Protoc. Exch..

[26] Corley R., Tinker P. (2003). Growth, flowering and yield. The Oil Palm.

[27] Ralph S.G., Yueh H., Friedmann M., Aeschliman D., Zeznik J.A., Nelson C.C., Butterfield Y.S.N., Kirkpatrick R., Liu J., Jones S.J.M. (2006). Conifer defence against insects: Microarray gene expression profiling of Sitka spruce (*Picea sitchensis*) induced by mechanical wounding or feeding by spruce budworms (*Choristoneura occidentalis*) or white pine weevils (*Pissodes strobi*). Plant Cell Environ..

[28] Ferry N., Stavroulakis S., Guan W., Davison G.M., Bell H.A., Weaver R.J., Down R.E., Gatehouse J.A., Gatehouse A.M.R.R. (2011). Molecular interactions between wheat and cereal aphid (*Sitobion avenae*): Analysis of changes to the wheat proteome. Proteomics.

[29] Rivera-Mendes Y.D., Cuenca J.C., Romero H.M. (2016). Physiological responses of oil palm (*Elaeis guineensis* Jacq.) seedlings under different water soil conditions. Agron. Colomb..

[30] Azzeme A.M., Abdullah S.N.A., Aziz M.A., Wahab P.E.M. (2016). Oil palm leaves and roots differ in physiological response, antioxidant enzyme activities and expression of stress-responsive genes upon exposure to drought stress. Acta Physiol. Plant..

[31] Durrant W.E., Dong X. (2004). Systemic acquired resistance. Annu. Rev. Phytopathol..

[32] Rejeb I., Pastor V., Mauch-Mani B. (2014). Plant responses to simultaneous biotic and abiotic stress: Molecular mechanisms. Plants.

